# Cultural factors and senior tourism: Evidence from the Chinese context

**DOI:** 10.3389/fpsyg.2022.1030059

**Published:** 2022-11-23

**Authors:** Jinmei Tuo, Renli Deng, Ming Tao, Zucai Xu, Yan Peng, Yushuang Linghu, Shiming Huang, Changyin Yu

**Affiliations:** ^1^Department of Nursing, Affiliated Hospital of Zunyi Medical University, Zunyi, China; ^2^Department of Neurology, Affiliated Hospital of Zunyi Medical University, Zunyi, China; ^3^The Collaborative Innovation Center of Tissue Damage Repair and Regeneration Medicine of Zunyi Medical University, Zunyi, China

**Keywords:** older population tourism, tourism motivation, culture influence, older population tourism products, tourism experience

## Abstract

Recently, numerous studies have focused on tourism among the older population. Of them, most reported on status analysis, tourism motivation, and tourism model, to name a few; however, there was a lack of comprehensive synthesis and analysis of the motivation, influencing factors, policy impact, and other factors of older tourism. Thus, this study conducted various keyword searches among both English and Chinese publications. We found that older population’s tourism is affected by various factors, such as travel expense, physical condition, the length and distance of a trip, and cultural influence. The results provide a reference for the development and implementation of tourism among the older population.

## Introduction

**Figure 1:**
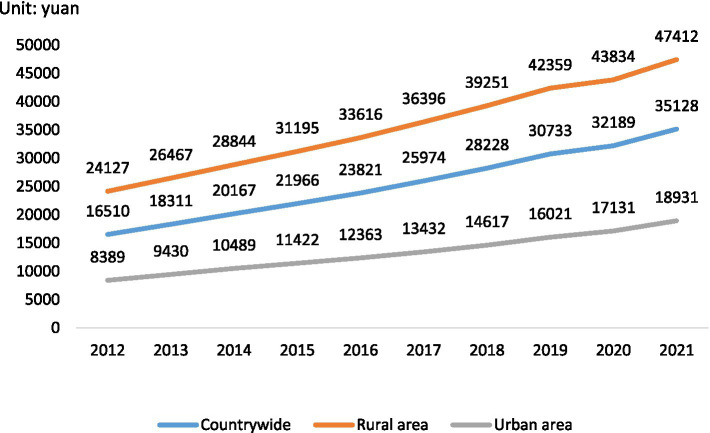
Disposable income *per capita* from 2012 to 2021. Data compiled by National Bureau of Statistics of China (2021).

According to World Bank data, the proportion of the population aged 65 and over in China doubled from 6.81% in 2000 to 12.41% in 2021 (The World Bank, 2021). Due to rapid economic development (Figure 1), great changes have taken place in China over the past few decades. Urbanization has increased rapidly, with the number of cities increasing from 193 in 1978 to 687 in 2020 (Li and Li, 2022). Further, the proportion of the urban population increased from 26.44% in 1990 to 63.89% in 2020, with an average annual growth rate of 1.09%. Compared with 2010, the proportion of the urban population increased by 14.21% (China Statistics Press, 2021; National Bureau of Statistics of China, 2021). Moreover, according to data from the China Statistical Yearbook 2021, the number of domestic tourists increased from 4.435 billion in 2016 to 6.006 billion in, 2019 (National Bureau of Statistics of China, 2021). Under the influence of the coronavirus disease, 2019 (COVID-19), there were still 2.879 billion tourists in 2020 (National Bureau of Statistics of China, 2021). With continuous economic improvement, people increasingly choose to travel.

Meanwhile, population ageing has become a general trend in human development. The ageing population is an increasingly severe issue and presents problems that have gradually affected all aspects of society, the economy, culture, and life (Huang and Chen, 1999). As a developing country, China has had a very obvious trend of population ageing since the 1970s. Table 1 shows the total number of the older population (over 60 years of age), according to prediction results for 2015–2050. In five-year intervals, the older population will be 232, 275, 333, 398, 446, 463, 473, and 498 million, respectively (Chen et al., 2018). Exploring the positive effects of an ageing society is conducive to coping with the severe challenges posed by population ageing. In this context, tourism in an ageing society should be studied in depth from many aspects, and many methods are applicable to this (Sedgley et al., 2011).

**Table 1:** Forecast results of the Chinese older population from 2015 to 2050.

	60–64	65–69	70–74	75–79	80–84	85–89	>90	Total
2015	7,890	5,583	3,781	2,840	1871	885	388	23,237
2020	7,486	7,509	5,134	3,254	2,222	1,234	638	27,478
2025	9,911	7,124	6,908	4,420	2,546	1,464	928	33,302
2030	11,608	9,433	6,552	5,949	3,460	1,679	1,168	39,849
2035	10,916	11,048	8,678	5,641	4,661	2,281	4,380	44,606
2040	8,941	10,389	10,165	7,475	4,418	3,077	1795	46,258
2045	9,268	8,510	9,558	8,755	5,856	2,915	2,395	47,258
2050	11,660	8,824	7,830	8,232	6,861	3,867	2,545	49,818

With changing times, as far as tourism is concerned, changes in traditional concepts have gradually diversified consumption by the older population, who increasingly participate in tourism activities, thus forming a tourism market for this group with a certain scale. Leisure and tourism are important ways to improve the happiness of the older population, which is one of the important characteristics of successful population ageing (Wang and Luo, 2014). At the same time, increases in tourism consumption are occurring more rapidly, and the consumption scale is immense. Older population tourism has, thus, become an important part of the tourism industry. The development of older population tourism is an important measure in dealing with the ageing of the Chinese population. Combining tourism and the successful ageing field when studying the tourism behavior of the older population is an innovative way to explore the ageing problem from an interdisciplinary perspective. According to statistics from the Ministry of Culture and Tourism of the People’s Republic of China (PRC, 2019), older adult tourists represent about 30% of all tourists. Xia (2015) showed that the older population are most attentive to the scenery when they travel, followed by cost, traffic, and, finally, local safety. Their tourism finances generally depend on their savings, and they rarely rely on other sources of support. Further, the older population mainly derive information about older population tourism from family members and friends, followed by advertising from television networks and travel agencies. In tour groups, tour guides make arrangements for transportation, board, and lodging and provide tourism services to the older population (Hu, 2022). The main hindrances to their travel are their physical condition and age (Xia, 2015). Moreover, the older population generally believe that their happiness will be greatly improved after travelling.

Most recent studies have focused on the older population’s health tourism (Wang and Luo, 2014; Chen et al., 2022). The older population can choose from a variety of tourism methods, such as overseas travel, high-end cruise vacation, and customized tourism (Hu, 2022). The form and content of their tourism choices are becoming increasingly diverse. Furthermore, the older population’s motivations are different, and the relevant study results are different (Wang, 2022). Medical and health tourists can also receive medical services while touring (Chen et al., 2022). The main purpose of health and wellness tourism is maintaining or enhancing physical health, while nourishing their minds; meanwhile, nature tourists typically pursue spiritual relaxation or mental health, expanding their vision and connecting better with the spiritual realm (Chen et al., 2022). Although previous studies have discussed the motivation for the older population to choose tourism in terms of health (Song, 2021), culture (Huang et al., 2021), and the economy (Gu and Wu, 2003), few studies have systematically reviewed the tourism motivation of the older population. The development of older population tourism in China is still relatively slow, clearly lagging in terms of the needs of older tourists. The overall promotion and coordinated development of Chinese older population tourism are imperative. At the same time, studying the development of ageing tourism not only serves to explore and enrich the older tourism industry but also provides relevant guidance and support for the current development of the older population tourism industry in China.

Therefore, the purpose of the present study was to systematically summarize the current situation and factors influencing tourism in the older population, confirm the motivations that affect the older population’s participation in tourism activities, and propose feasible suggestions for standardization of the older population tourism market. The results will provide a reference for the development and implementation of older population tourism.

## Materials and methods

Various keyword searches were conducted using a range of both English and Chinese publications. We used ‘old population tourism’, ‘tourism motivation’, ‘culture influence’, ‘travel behavior’, ‘tourism experience’, and ‘tourism products’ as the subject words in Chinese and English retrieval. To retrieve English articles, PubMed, Web of Science, MEDLINE, and Google Scholar were used, and to retrieve Chinese articles, CNKI and Wanfang were used. We then integrated, analyzed, and summarized literature that met the inclusion criteria of this study.

## Results

### Characteristics of tourism in the older population

**Figure 2:**
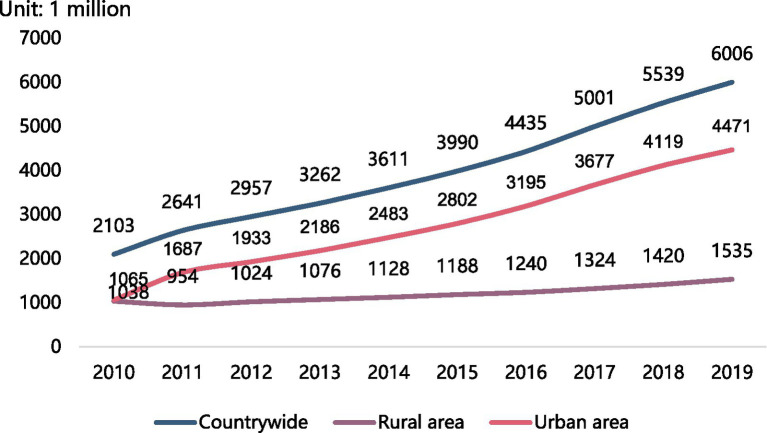
Domestic tourists in China. Data compiled by National Bureau of Statistics of China (2021).

During retirement, the older population develop certain economic foundations and abundant leisure time, making the concept of enjoying life increasingly popular. Nimrod’s (Nimrod, 2008) study shows that tourism plays a central role in the post-retirement reality. Active travel has become an important way for older adults to improve their quality of life; it is also becoming a common leisure and entertainment activity, as well as a common lifestyle for the older population (Wang and Luo, 2014). A survey showed that the older population tourism market has huge market potential, with considerable expansion space and development prospects. At the same time, the cost of older population tourism showed a continuous growth trend (Figure 2). Previous studies have shown that compared with younger people, the older population tend to take shorter trips, are more interested in walking, and travel less frequently (Böcker et al., 2017). Moreover, the older population is a special group. Among the older population, the process of tourism involves quality of life and consumption ability. Tourism is both physically and psychologically different for them compared to the general population, so tourism services should be offered in line with the characteristics of the older population. Moreover, retirement may affect the older population’s travel plans as they may take care of their grandchildren, engage in volunteer work, or have family responsibilities (Mifsuda et al., 2017). We must note that physical decline with ageing may also lead to a decline in life satisfaction for older adults, which can affect their travel plans. These hindrances to travel may be further exacerbated by the traditional culture of China.

Due to the influence of the one-child policy, many older populations have superior material living conditions but are more likely to feel lonely because their children must work. Many older populations choose to travel to enrich their spare time and feel mentally satisfied. In the process of travelling, they can enjoy natural sceneries, broaden their horizons, increase their knowledge, enjoy life, and eliminate the loneliness caused by the desolation of their family (Zhao et al., 2013). At the same time, because most older populations are retired and stay at home, they have a lot of flexibility in deciding when to travel (Liu et al., 2021).

The older population are also prone to nostalgia and prefer tourism environments conducive to physical and mental health (Zhang and Li, 2009). Moreover, they like to stay away from the hustle and bustle of the city and prefer quiet and beautiful landscapes. In addition, the older population are deeply influenced by Chinese history and traditional culture, which they have a deep understanding of, and have a special homesickness complex. Red nostalgia (a fondness for reminders of communist history in historically communist countries like China), landscape scenery, and health care travel have become the preferred travel modes for the older population (Zhang and Li, 2009). Currently, travel among the older population tends to be more diversified than before.

According to a previous study’s results, income level, offspring’s attitude, scenic environment, medical facilities, climate environment, and education level have a significant positive impact on the older population’s willingness to travel (Zhang et al., 2021). Meanwhile, age, family structure, and health status have significant negative effects (Zhang et al., 2021). Therefore, it is very important to actively guide the older population’s children to pay attention to how the older population live while ageing and provide support for their tourism pension activities in terms of encouragement and capital. The life cycle not only affects the travel obstacles perceived by the elderly, but also affects the attitude of the elderly toward travel, destination selection decisions, and travel activities. Therefore, it is necessary to subdivide the elderly leisure market regarding generation and life cycle (Chen and Shoemaker, 2014). Moreover, given that older population tourism may be seen as an unnecessary luxury, it is important to alter negative perceptions around the idea of using pensions for tourism and to improve publicity in this regard.

### Influence of culture on tourism in the older population

Previous research shows that asymmetric and nonlinear effects have been observed in the development of tourism in most destination countries (Lee et al., 2022). Some researchers suggested that, based on a sense of belonging and identity, people are more likely to accept familiar cultural programs (Pastina and Straubhaar, 2005). Due to the influence of their traditional culture which can be traced back to ancient times, Chinese people have a special attachment to their home, which can also be traced back to ancient times. Therefore, when there is a large travel distance between the departure site and destination, tourists’ perception of the destination is affected by cultural proximity and the physical environment (Pastina and Straubhaar, 2005). Especially due to the impact of COVID-19 and the development of social networking culture, accessing tourism resources has become more difficult for the older population. It is particularly important to improve tourism operators’ quality of catering to the older population, as well as their education and training in basic medical knowledge.

In addition, because of the development of the Internet, traditional tourism advertising has been migrating to online platforms. However, the older population have difficulty understanding how to use the Internet and cannot effectively obtain some tourism information. Jalilvand et al. (2012) showed that there was a significant connection between destination image and tourism intention, so older tourists may limit their tourism destination options because they have no clear impression of certain destinations. It is, therefore, necessary to help them make full use of the Internet (based on regional cooperation and industrial integration), adjust the mode of industrial development, improve the quality of tourism human resources, and comprehensively intensify the scale and benefits of the development of tourism in the older population (Guan et al., 2022).

Broader cultural factors influence tourism-related decisions. Different evaluation dimensions of culture will produce different results. For example, a large cultural distance is more likely to bring new experiences to tourists (Huang et al., 2021). In addition, a previous study in Japan suggests that an optimal point of the cultural, administrative, geographic, and economic distance for purchasing behavior of tourists exists (Li and Katsumata, 2019). The opportunities for the older population to travel are greatly affected by the fact that the national culture and needs of elderly tourists vary across countries; the higher the income and the longer the leisure time, the stronger the desire to travel (Huang et al., 2021). Therefore, the older population have the desire and ability to travel when they have both time and money. At the same time, because the incomes of most of the older population do not increase significantly after retirement, they mostly tend to choose domestic destinations and package tours (Javalgi et al., 1992). Older populations with higher incomes and in higher social positions are more likely to buy tourism products and travel to show their achievements and meet their demand for status. Under the influence of culture, the older population pay more attention to satisfaction of diversity and enjoyable experiences and are more inclined to travel to meet the needs of rest, relaxation, self-improvement, and increased life experience (Huang et al., 2021).

### Policy support for tourism in the older population

According to the State Council of the PRC:

The 14th five-year plan and the outline of the long-term goals for 2035, we should aim to improve the modern cultural system, further develop public tourism and wisdom tourism, innovate the tourism product system, improve the tourism consumption experience, strengthen the integration of regional tourism brands and services, promote red tourism, cultural heritage tourism, innovative development of tourism performance, etc., and improve the quality of vacation and leisure, rural tourism, and other services. Additionally, it is necessary to improve tourism infrastructure and distribution systems, establish tourism service quality evaluation systems, and standardize online tourism management services (The State Council of the PRC, 2021).

Many relevant measures have been introduced across many local governments to promote tourism for the older population. For example, strengthening the supply of cultural services for the older population; in-depth implementation of basic aged care services in the older population; and medical treatment, tourism, cultural activities, leisure, transportation, going out, and so forth, have been written into the 14th five-year plan for the development of the elderly cause and the older population care service system by Guizhou Province (Gazette of Guizhou Provincial People’s Government, 2022). Beijing has established the ‘Beijing Smart Tourism Map’ and explored a new mode of elderly service. Beijing has also adopted a cultural tourism reception base for the elderly. This guides the older population in understanding the environmental conditions of scenic spots, so that they explore and compare tourist attractions and red scenic spots without leaving home as a reference for the next step towards travel (Beijing Municipal Bureau of Culture and Tourism, 2022). The Shanghai Municipal People’s Government proposes that to promote tourism development among the older population, their tourism should be considered an important part of tourism overall. Red tourism, cruise tourism, health tourism, and other tourism formats suitable for the older population should be vigorously developed. Additionally, it is necessary to develop more tourism products suitable for the older population online and offline and create a characteristic tourism model. The municipal government aims to eliminate barriers and strengthen the management of tourism infrastructure, such as scenic spots and hotels, improve the level of humanized services, and create a safe and convenient tourism environment for the older population. They further aim to support tourism enterprises to actively improve the standards of tourism products and services for the older population (Shanghai Municipal People’s Government, 2020). According to the official website of the Tianjin Municipal People’s Government, they plan to make full use of the Centre of Tianjin Tourism’s new media data and the consulting network of Tianjin tourism, as well as Weibo and WeChat, to carry out online and offline interactive marketing and shape the tourism brand of the Beijing-Tianjin-Hebei region. They will promote the greater integration of the health industry and tourism. In view of the new demands of health tourism, such as health preservation, ageing care, and healthy ageing, they will vigorously develop traditional Chinese medicine health tourism, continue to promote health tourism projects featuring traditional Chinese medicine, strengthen the management and guidance of the construction of a traditional Chinese medicine health tourism demonstration base, and explore an innovative mechanism for the integrated development of traditional Chinese medicine tourism. At the same time, they will implement a preferential policy of reducing or exempting ticket prices for the older population (Tianjin Municipal People’s Government, 2020).

According to Zhou (2010), the older population have both the demand for travelling and the financial ability to meet this demand. Thus, the demand for older population tourism is strong. Therefore, with increased ageing, the older population tourism industry has begun to be favored by increasing numbers of the older population (Li and Li, 2022). Older population tourism needs not only the intelligent development of enterprises but also the strong support of local governments.

### Challenges of tourism in the older population

First, supply and demand within the older population tourism market do not match, and there are even some disjointed situations. The design and arrangement of some tourism products provided by tourism agencies are not scientific and reasonable enough to take the characteristics of the older population into account (Zhou, 2010). The products are insufficiently developed, and service quantity cannot meet market demand. Despite these issues, the older population hope to obtain high-quality tourism experiences. The mismatch between supply and demand is a result of the contradiction between the pursuit of physical and mental health for the older population and the pursuit of economic interests of tourism enterprises. However, in the current tourism market of China, tourism enterprises have not put forward enough special health tourism products targeting the older population, the service quality is mixed, and market satisfaction is generally not high. As a result, it is difficult to integrate the tourism products that have been developed with the older population. The older population have certain economic advantages and relatively large amounts of free time. However, influenced by Chinese culture, they have developed habits of diligence and thrift. In terms of tourism consumption, most of them primarily choose economic tourism, tending to be practical and rational. Moreover, the older population have relatively high requirements for safety assurance and service quality in the overall tourism process, while the overall tourism market for the older population is relatively homogeneous. The available tourism products are similar in content and few in quantity, which lacks attractiveness for the older population. Tourism companies see the potential of the older population tourism market, but the risk is large. Some tourism companies even use exaggerated publicity to attract the older population while increasing self-payment projects in the process so that older tourists feel cheated. Such behavior will seriously disrupt the normal tourism market. Therefore, the development of the older population tourism market needs not only the participation of tourism companies, but also the standardized management and guidance of relevant departments (Zhou, 2010).

Second, the ‘sunset red tour’, ‘silver hair tour’, ‘filial piety to parent’s tour’, and other tourism projects active in the China tourism market in recent years have increasingly attracted attention to older population tourism. However, they also have some drawbacks, such as difficulty in transportation transfer, managing the different needs of the older population with different cultural interests, nutrition matching of different dietary habits, differences in consumption levels, as well as the lack of carefully thought-out scenic spots and times for activities. All of these have an influence on the older population’s tourism experience (Song, 2021). Although the older population’s demand for tourism has been increasing, the response of travel agencies has not been warm. This is often closely related to the above reasons. Due to the influence of their generation’s cultural background, the older population in the current period pay more attention to health preservation, health care, medical treatment, visiting relatives, making friends, visiting friends, and so forth (Hu and Li, 2022). Younger people travel to mountainous and rural areas to create memories that they can reminisce about in their later years and, in these later years, they hope to visit the cities that they had once worked in again. Moreover, many older populations hope to experience the red culture, accept the edification of red culture, walk the path of revolutionary martyrs again, and experience the hard-won fruits of revolution.

Third, with increasing age, the physical and physiological functioning of the older population changes and vision, hearing, and touch also gradually deteriorate. For example, slow response times, poor physical flexibility, hyposthenia, nervous system degradation, anorexia, weight loss, and memory loss begin to appear (MacIntosh et al., 2000; McLean and Le Couteur, 2004; Robbins et al., 2019). Psychologically, the older population have a lower ability to accept new things and are prone to feel inferior, lonely, and lost; they are also prone to fear, anxiety, depression, sensitivity, and stubbornness (Robbins et al., 2019).

Finally, in recent years, due to its rapid development, the Internet has been widely used in entertainment, social networking, medical care, and other fields, affecting all aspects of society. A study has shown that Internet use by the middle-aged and older population is an important factor affecting their happiness in life (Wen and Ding, 2022). Therefore, middle-aged and older populations are actively encouraged to develop Internet skills, which can enrich their social, entertainment, cultural and medical life, as well as other areas of their lives. Further, this could improve their relationships with their offspring, relieve their anxiety and depression, and improve their physical and mental health. However, the Internet is a new thing for the older population. Due to their cultural background, there is no suitable learning path for them, which creates many difficulties in the popularization of the Internet.

To sum up, the older population’s tourism is affected by many factors, such as travel expense, physical condition, the length and distance of a trip, and cultural influence. In the future, older population tourism should not only focus on the development and innovation of tourism products, but sociological factors, such as culture and education, will also need to be researched. Furthermore, it is necessary to study how physiological and psychological characteristics affect the older population’s tourism motivation.

### Proposals for the older population tourism industry in China

Tourism consumption behavior is an unconventional, comprehensive, and experiential behavior that is different from general consumer behavior and is extremely susceptible to external factors. Huang and Cui (1994) regarded household tourism behavior as a high-level consumption behavior within the scope of spiritual and cultural consumption. With increases in discretionary funds, the older population’s attitudes to consumption have also begun to change, being no longer ‘behind the times’, no longer satisfied with basic material necessities, but eager for more colorful leisure and entertainment activities. They are willing to go out to experience nature, visit relatives and friends, travel around, and gain spiritual pleasure while acquiring knowledge. Hence, we propose the following suggestions for the development of older population tourism in the present study.

First, we recommend developing multi-level, diversified, and high-quality tourism products for the older population that consider their physiological and psychological characteristics to meet their needs in a deeper and more detailed way. A tourism brand for the older population should be built and distinctive tourism service modes, such as cultural, landscape, and health tourism, should be introduced to induce potential demand in the older population. At the same time, the older population are likely to feel lonely because of the deaths of their friends. Therefore, older population tourism should not only focus on the older population but also introduce a tourism mode that is suitable for their offspring to travel with them, thus increasing the amount of time that older population spend with their families.

Second, the design of tourism products should reasonably plan the tourism route, combine long-distance tourism with short-distance tourism, integrate tourism resources to suit the needs of the older population in their province from their own perspective, and create a themed tourism route combining health and care. The travel period should not be too long, slow down the pace of tourism, and pay attention to safety and tourism services. Tourism products should be effectively communicated to older population, and service quality should be improved according to the travel experience of and feedback from older tourists. At the same time, targeted training should be provided to older population tourism practitioners on issues related to the older population, such as physiological and psychological changes and nutritional input, to improve humanized services and enable the older population to have better experiences and happiness through tourism. In addition, publicity should consider the characteristics of the older population when tourism products are developed and try to use eye-catching words and colorful patterns. We recommend making full use of the rapid development of ‘Internet +’, adopting online and offline publicity modes, and making good use of traditional publicity methods, such as radio, television, and newspapers, to attract more older tourists. We also recommend actively developing practical and easy-to-use tourism apps according to the special requirements of the older population, which could comprehensively add regional culture, local characteristics, customs, and folkways to create a brand-new older population tourism platform and service products. At the same time, in combination with relevant information from medical institutions, to ensure their safe travel, timely and effective emergency services can be provided for the older population should the need arise (Li et al., 2020). Moreover, this should be combined with national preferential policies, and a series of tourism products should be designed that conform to the needs of the older population, depending on personal hobbies, nostalgia, homesickness complex, health care, cultural experiences, and so on. Then, the older population should be informed of the related tourism details with pictures and videos that cater to their abilities before travel begins.

Finally, older population tourism needs support and supervision from the government to create a good travel environment, have a clear plan for older population tourism, clarify the development direction and orientation of the older population tourism market, establish a sound social welfare system for the older population, and introduce relevant preferential policies for older tourists. Due to the influence of traditional Chinese culture, the older population mostly adhere to values of diligence and thrift. Therefore, traditional cultural values also hinder the older population from travelling. However, in the present era, the world has begun to move closer to integration of Chinese and Western cultures. Affected by this, many older populations have begun to use tourism to enrich their experience. Most older populations have a strong willingness to participate in leisure activities and are willing to invest their money, time, and energy in improving their sports skills and activity participation (Long and Wang, 2013). According to the local tourism situation and the characteristics of the older population’s economic resources for travel, preferential policies for off-season tourism can be introduced to stimulate the older population’s tourism motivation so that their tourism experience meets their expectations (Luo and Li, 2021). At the same time, they should improve the software and hardware facilities of older population tourism, such as barrier free thoroughfares and barrier free equipment construction at various scenic spots, hotels, restaurants, and toilets, to create a good tourism atmosphere for older population tourism. In addition, while developing, opening up, and supporting older population tourism, all government departments should play a leadership role, strengthen the supervision of older population tourism enterprises, effectively safeguard the legitimate rights and interests of older tourists, and improve their tourism experience and happiness index.

## Discussion

The acceleration of the aging of society has had a wide impact on society in general, the economy, and culture (Huang and Chen, 1999). In recent years, with the improvement of the older population’s economic and health levels, they are eager to obtain a more meaningful elderly life, including participating in tourism and receiving life-long education (Wen and Ding, 2022). In this regard, tourism has become one of the important ways to enrich the lives of the older Chinese population (Wang et al., 2017). The older population pay more attention to enjoying the scenery when they travel, followed by cost, traffic, and, finally, local safety. However, the older population mainly derive tourism information from family members and friends, followed by advertisements by TV networks and tourism agencies. Most older populations choose to travel in groups, and the main factors that hinder tourism in older populations are their physical condition and age (Xia, 2015). Therefore, in designing tourism products for the older population, their particular needs should be fully considered and tourism routes should be designed according to local characteristics, natural scenery, and different cultural characteristics. Additionally, effective publicity should be carried out using a combination of both online and offline modes and the training of older population tourism practitioners should be strengthened to improve the older population’s tourism experiences. Furthermore, emergency care and safety for the older tourism population should be guaranteed.

Moreover, culture has a great impact on tourism among the older population. Influenced by their traditional culture, Chinese people, especially the older population, have long had a special attachment to their home. Cultural differences within China also produce different results (Gu and Wu, 2003). Therefore, tourism products for the older population that consider this background can still be further developed. Through tourism, the older population can not only deepen communication and exchange with their offspring, but also achieve the goals of rest and relaxation. Thus, the tourism experience of the older population can be improved. Moreover, the impact of the rapid development of the networking culture on the older population should not be ignored. Helping them to learn relevant knowledge and operating skills can also effectively improve how they obtain tourism information. Older population tourism practitioners should realize that tourism consumption behavior is different from ordinary consumption behavior and, therefore, satisfy the needs of the older population in a more in-depth and comprehensive way.

The development of tourism for the older population cannot be separated from support by state and local governments. From the state level to all local governments, many relevant measures for the older population have been introduced. These will promote older population care service systems that integrate ageing services, medical care, tourism, cultural activities, and leisure.

However, the older population tourism industry also faces many challenges, such as unbalanced supply and demand, the unreasonable design and arrangement of tourism products, and that the characteristics of the older population are not taken into consideration (Zhou, 2010). Furthermore, service quality does not conform to market demand. Existing tourism products are not attractive to the older population, as most of them are similar in content, and their numbers are insufficient, so the service quality cannot be guaranteed, which reduces the older population’s tourism experience. In addition, the influence of age and cultural background on the older population cannot be ignored. To a certain extent, the older population also have a need to rekindle previous memories and visit their relatives and friends. Therefore, older population tourism design should also fully consider these needs. Although the demand for older population tourism has risen in recent years, the response of tourism agencies has not been enthusiastic. The reasons are often closely related to the aforementioned factors.

The present study discusses the motivational and influencing factors of older population tourism and proposes some suggestions based on these factors. In the future, there are still many issues to be discussed, for example, the impact of ethnic differences and how education affects older population tourism. Further research on older population tourism is particularly important in healthy ageing.

## Conclusion

There are still many problems in tourism for the older population. In China, older population tourism to learn, eventually pursue life of continuity and respect, and discover the changes of country with a high sense of pride and patriotism exist (Hsu et al., 2007). It is necessary to consider personal factors, such as physical functioning and health condition, as well as social factors such as culture, habits, and safety when developing and designing tourism products. In addition, when designing and propagating tourism products, we need to conform to the needs and characteristics of the older population. We also require guidance, assistance, and support from different levels of government.

## Author contributions

JT: writing original draft. SH and CY: supervision. RD and MT: conceptualization. ZX: methodology. YP: investigation. YL: investigation and methodology. All authors contributed to the article and approved the submitted version.

## Funding

The present study was supported by the Guizhou Provincial Science and Technology Plan Project Contract Number: Qiankehe Support (2021) General 446.

## Conflict of interest

The authors declare that the research was conducted in the absence of any commercial or financial relationships that could be construed as a potential conflict of interest.

## Publisher’s note

All claims expressed in this article are solely those of the authors and do not necessarily represent those of their affiliated organizations, or those of the publisher, the editors and the reviewers. Any product that may be evaluated in this article, or claim that may be made by its manufacturer, is not guaranteed or endorsed by the publisher.
